# Is the BCG Vaccine Safe for Undernourished Individuals?

**DOI:** 10.1155/2012/673186

**Published:** 2012-04-10

**Authors:** Larissa Lumi Watanabe Ishikawa, Larissa Camargo da Rosa, Thais Graziela Donegá França, Raphael Sanches Peres, Fernanda Chiuso-Minicucci, Sofia Fernanda Gonçalves Zorzella-Pezavento, Alexandrina Sartori

**Affiliations:** Departamento de Microbiologia e Imunologia, Instituto de Biociências, Universidade Estadual Paulista (UNESP), 18618-970 Botucatu, SP, Brazil

## Abstract

Cellular immunity is critical for protection against tuberculosis, but its integrity is compromised during undernutrition. The present study was designed to evaluate if the attenuated mycobacterium BCG is a safe vaccine for undernourished individuals. An experimental model of undernutrition was established by subjecting BALB/c mice to dietary restriction. These animals received 70% of the amount of food consumed by the healthy control group and exhibited physiological alterations compatible with malnutrition, including body weight loss, reduced levels of triglycerides and glucose, and reduced lymphocyte numbers. Undernourished mice were immunized with BCG, and the mycobacterial loads in lymph nodes, spleen, liver, lungs, and thymus were determined. A much higher proportion of undernourished mice exhibited bacterial dissemination to the lymph nodes, spleen and liver. In addition, only undernourished animals had bacteria in the lungs and thymus. Concomitant with higher mycobacterial loads and more widespread BCG dissemination in undernourished mice, production of TNF-*α*, IFN-*γ*, and IL-10 was also diminished in these mice. Taken together, these results indicate that BCG infection is more severe in undernourished mice. Whether a similar phenomenon exists in undernourished children or not remains to be thoroughly investigated.

## 1. Introduction

Undernutrition is one of the major causes of mortality and morbidity worldwide. Latest estimates indicate that over 800 million people in the world are undernourished [1]. Poverty and lack of adequate sanitary conditions contribute to a higher prevalence of malnutrition in underdeveloped countries. Protein-energy malnutrition (PEM) is the most common type of undernutrition and is caused by deficiencies in protein or macro- and micronutrients such as vitamin A, vitamin E, vitamin B6, folate, zinc, iron, copper, and selenium [2]. Inadequate energy intake leads to various physiological deficiencies, such as growth deficiencies and loss of fat, muscle and visceral mass, and has deleterious effects on the immune system [3]. Undernutrition has been linked to many deficiencies of the innate immunity as decreased lysozyme production by monocytes and polymorphonuclear cells (PMNs), depletion of complement components, and impairment of macrophage functions [4]. Adaptive immunity is also compromised during nutritional depletion. PEM impairs, for example, cytokine production, T-cell function, and the ability of lymphocytes to appropriately respond to these cytokines [5].

The most immediate consequence of undernutrition is increased susceptibility to infections. Tuberculosis (TB) is a disease caused by *Mycobacterium tuberculosis* that has historically been known to be influenced by undernutrition. It is the major cause of morbidity and mortality in developing countries where malnutrition is also prevalent [6]. It is also well established that the cellular immune response, whose efficacy is compromised during undernutrition, is required to protect against TB. The only available vaccine against TB is the attenuated *Mycobacterium bovis* Bacillus Calmette-Guérin (BCG) that is recommended by the World Health Organization for all infants under one year of age. Around 100 million newborn children are given this vaccine, and the global vaccine coverage is estimated to be 80% [7]. Regardless, large numbers of well-documented trials show extensive variation in the protective efficacy of BCG, ranging from 0 to 80% [8]. Although the overall efficacy is low, most studies agree that BCG protects against disseminated disease in newborns and children but that this immunity wanes with age, resulting in no or insufficient protection against adult pulmonary TB [9, 10]. For this reason, there is great interest in developing new vaccines to prevent TB infection. A number of alternative living and nonliving TB vaccines, including genetic vaccines, are currently being investigated [11–13]. For example, DNAhsp65 is a genetic vaccine containing the gene of mycobacterial 65 kDa heat shock protein that has also been used in prime-boost protocols as an attempt to improve BCG efficacy. Safe and effective heterologous prime-boost regimens that augment BCG or recombinant BCG immunization are considered the most realistic strategies for TB control [10]. We previously reported that both DNAhsp65 and BCG could prime newborn mice for stronger immune responses to DNAhsp65 boosters at the adult stage [13]. Prime-boost strategies combining these two vaccines were also able to protect well-nourished mice and guinea pigs against TB infection [14, 15]. More recently, we demonstrated that the antibody induction by the same genetic vaccine (DNAhsp65) was abrogated in animals submitted to dietary restriction [16]. We also observed that immunization with formalized *Staphylococcus aureus* protected well-nourished mice but not undernourished ones from lung injury in methicilin-resistant *S. aureus* infection [17].

The present study was designed to compare BCG dissemination and load between well-nourished and undernourished mice.

## 2. Materials and Methods

### 2.1. Animals

 Four- to five-week-old isogenic female BALB/c mice were housed in plastic cages with white wood chips for bedding, free access to filtered water, and conditions of controlled lighting (12 h light/12 h dark cycle) and temperature (23 ± 2°C). After weaning, mice were acclimated on standard chow for 10 days (Purina, Paulínia, SP, Brazil). This animal chow is considered adequate for mice and it is officially approved by the Brazilian Ministry of Agriculture (SP-0311730758). Animals were manipulated in compliance with the ethical guidelines adopted by the Brazilian College of Animal Experimentation, and the experimental protocol used in this work was approved by the local ethics committee (19/08-CEEA).

### 2.2. Experimental Design

We initially analyzed physical, hematological, biochemical, and immunological parameters associated with 10 days of undernutrition. For this, mice were allocated into two experimental groups: the control group (normal) and the undernourished one (restricted). The first one was fed *ad libitum* and the second one received only 70% of the amount of food consumed by the control group. Bacterial loads and cytokine production were then compared in normal and undernourished mice intradermally inoculated with BCG (10^5^ CFU) at the base of the tail. Immunization of the undernourished group was delivered at the day 10 after the beginning of dietary restriction. The evaluations were performed 25 days after the beginning of food restriction. In the aforementioned evaluations, both experimental groups were housed in a ventilated caging system. To test the effect of household contact, normal and undernourished nonimmunized mice were also housed in conventional open cages, in the same room of immunized animals, before determination of bacterial loads in different organs.

### 2.3. Hematological and Biochemical Parameters

 Blood samples were collected by retro-orbital puncture and directly examined for hematological analysis or centrifuged for sera separation. Total leukocyte numbers were determined after blood dilution in Turk's solution, and differential leukocyte count was performed in blood smear stained with eosin/methylene blue (Leishman's stain). Blood glucose level was measured with Prestige Smart System (Home Diagnostics, FL, USA). Total protein, albumin, triglycerides and cholesterol in serum samples were measured by biuret, bromocresol green, and enzymatic Trinder standard methods, respectively (Labtest Diagnóstica SA, Belo Horizonte, MG, Brazil).

### 2.4. Cytokine Production

Murine spleens were removed, disrupted, and filtered in nylon cell strainers (Becton Dickinson, Franklin Lakes, NJ, USA). Splenocytes were resuspended in RPMI 1640 medium (Cultilab, Campinas, SP, Brazil) and then centrifuged (10 min, 1500 rpm, 4°C). Splenocytes were resuspended in RPMI medium supplemented with 10% fetal calf serum, 2 mM L-glutamine and 40 mg/mL gentamicin. Cells were plated at 5 × 10^6^ cells/mL in 48-well flat-bottomed culture plates (Nunc) and stimulated with 10 *μ*g/mL of recombinant heat shock protein 65-kDa (rhsp65) or 10 *μ*g/mL of concanavalin A (ConA) type IV-S (Sigma, St. Louis, MO, USA). Cytokine levels were evaluated 48 h later by enzyme-linked immunosorbent assay (ELISA) in culture supernatants using IFN-*γ* and IL-10 BD OptEIA Sets (Becton Dickinson) and TNF-*α* Duoset (R&D Systems, Minneapolis, MN, USA). The assays were performed according to the manufacturer's instruction.

### 2.5. Tissue Bacterial Loads

 Thymus, inguinal and popliteal lymph nodes, spleen, lungs, and liver from each mouse were collected and homogenized in 1 mL of saline. Next, 100 *μ*L of organ homogenates was plated in 7H11 mycobacteria agar and incubated at 37°C. After 30 days, *Mycobacterium bovis* colony forming units (CFUs) were counted.

### 2.6. Statistical Analysis

Statistical analyses were performed using SigmaPlot (Systat Software, CA, USA). Body weight was analyzed by paired *t*-test. All experiments related to BCG immunization were analyzed by one-way ANOVA parametric test or Kruskal-Wallis nonparametric test followed by comparative Dunn's test. Other results were analyzed by unpaired *t*-test. *P* values < 0.05 were considered statistically significant.

## 3. Results

### 3.1. Alterations Induced by Experimental Undernutrition

 Physical, hematological, biochemical, and immunological alterations possibly triggered by malnutrition were determined 10 days after the beginning of dietary restriction. At this time, body weight was already significantly lower in comparison to the normal group. Levels of triglycerides and glucose were also significantly below normal levels. Number of lymphocytes, PMNs, and monocytes in the undernourished group were all significantly lower than in normal animals. By this time, the production of IFN-*γ*, TNF-*α*, and IL-10 by spleen cells stimulated with ConA was similar in the two experimental groups. These results are shown in Table 1.

### 3.2. BCG Dissemination and Load in Undernourished Mice

**Figure 1:**
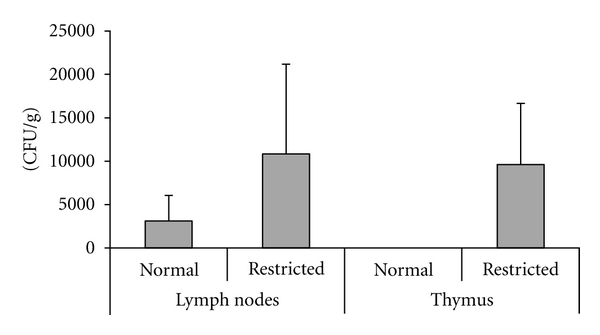
Effect of undernutrition on BCG dissemination. BALB/c mice were submitted to 10 days of dietary restriction and then immunized with BCG. Bacilli were recovered from the lymph nodes and thymus 15 days after BCG immunization. CFU values represent mean ± standard deviation of 6–10 animals.

 Fifteen days after subcutaneous BCG immunization, normal and undernourished mice were euthanized and bacterial loads in secondary lymphoid organs, liver, lungs and thymus were assessed. As expected, bacteria were not recovered from nonimmunized animals. However, striking differences were observed between normal and undernourished mice immunized with BCG. A much higher proportion of undernourished mice exhibited bacterial dissemination to lymph nodes, spleen, and liver, and only undernourished animals had bacteria in the lungs and thymus. Interestingly, BCG was recovered from undernourished but not from well-nourished household contacts (Table 2). Levels of bacilli recovered from lymph nodes and thymus of undernourished animals are illustrated in Figure 1.

### 3.3. Effect of Undernutrition and BCG on Cytokine Production

**Figure 2:**
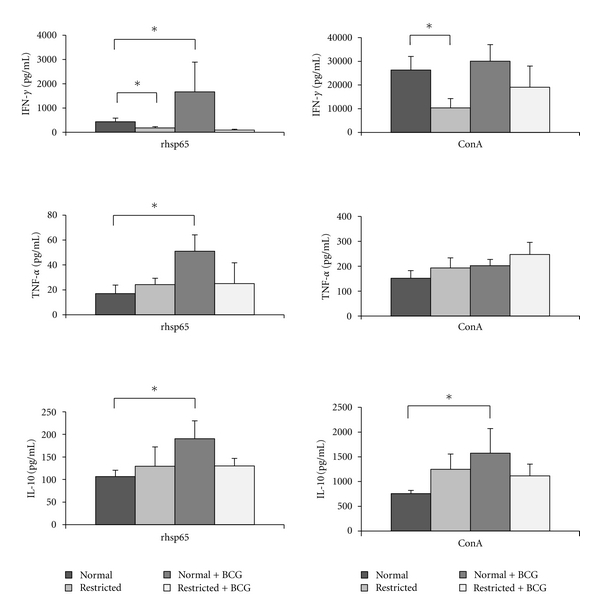
Effect of undernutrition on cytokine production by BALB/c mice immunized with BCG. Splenic cells were stimulated with rhsp65 or ConA, and IFN-*γ*, TNF-*α*, and IL-10 production were measured by ELISA. Evaluations were performed 25 days after the beginning of dietary restriction, that is, 15 days after BCG immunization. Values represent mean ± standard deviation of 6-7 animals. *P ≤ 0.05.

 Cytokine production was significantly affected by both undernourishment and BCG immunization. Spleen cells from undernourished mice produced less IFN-*γ* in response to rhsp65 stimulation than well-nourished animals. These cells also produced less IFN-*γ* after ConA addition. In normal mice, but not in undernourished ones, BCG immunization was associated with higher production of IFN-*γ*, TNF-*α*, and IL-10 by spleen cells stimulated with rhsp65 (Figure 2).

## 4. Discussion

 Experimental dietary restriction is being used to explore the effects of PEM on immunity and susceptibility to infectious agents [18]. However, no study to date has evaluated the effect of undernutrition on susceptibility to attenuated vaccines that could theoretically behave as virulent pathogens. We first characterized the malnourished state in a mouse model and determined its effect on BCG dissemination and load. The immunological alterations triggered by undernutrition and the effect of BCG on cytokine production in undernourished mice were also evaluated. BCG was intradermally injected after 10 days of dietary restriction when several changes were already evident. Body weight, number of total leukocytes, PMNs, and lymphocytes, and serum levels of glucose and triglycerides were all below normal levels in undernourished animals by this time point.

 Bacilli amount was determined in lymph nodes, spleen, thymus, and liver to compare the severity of BCG infection. The percentage of animals from which bacilli were recovered considering all evaluated organs was always much higher in the undernourished group. In addition, no bacteria were able to spread to the lungs and thymus in well-nourished mice, whereas bacilli recovery in the lungs and thymus of undernourished ones occurred in 50% and 82% of the animals, respectively. Another indication that BCG infection was more severe in undernourished mice was revealed by BCG loads in different organs. A significantly higher amount of bacilli was detected in the lymph nodes and thymus of undernourished immunized mice in comparison to their well-nourished counterparts. In contrast, there was no difference in BCG loads in the spleen, and liver of undernourished compared to well-nourished mice (data not shown).

 An additional experiment was performed to compare the effect of household contact between healthy and undernourished mice. No bacilli were recovered from well-nourished nonimmunized mice housed in the same environment as well-nourished BCG-immunized mice. In contrast, BCG was detected in the lymph nodes, spleen and liver of some undernourished nonimmunized household contacts, as shown in Table 2. This finding deserves further investigation and is relevant for undernourishment in household contacts, especially in the case of TB and attenuated viral and bacterial vaccines. Severe malnutrition is described as an important risk factor for the transmission of infections to children in household contact with adults with pulmonary TB [19].

To the best of our knowledge, this is the first report describing the effect of BCG in experimental undernutrition. Two points related to these findings deserve special attention. The first one relates to the efficacy of BCG and its potential to trigger disseminated BCG infection (BCGosis) in undernourished children. Children with severe combined immunodeficiency can develop BCGosis after BCG immunization as a result of their deficient immune system, which clearly illustrates that vaccinations should only be performed in immunocompetent individuals [20]. The second point relates to the immunological consequences of BCG presence in the thymus of undernourished mice. Only a few reports have analyzed mycobacterial colonization in the murine thymus. Nobrega et al. reported that colonization of the thymus by BCG was delayed and occurred at a time when bacterial load was already decreasing in other organs [21]. Our results showed that BCG colonized the thymus of undernourished immunized mice at an early time point, that is, 15 days after BCG inoculation, whereas no mycobacteria was detected in the thymus of well-nourished mice. Future investigations should clarify whether the presence of mycobacteria can influence thymic selection and consequently affect the course of mycobacterial or other infectious diseases. Interestingly, a recent report demonstrated that mycobacteria dissemination to the thymus rendered newly generated T cells tolerant to the invading pathogen [22].

Analysis of cytokine production 15 days after BCG immunization (25 days after the beginning of dietary restriction) indicated that splenocytes from malnourished mice were deficient in their ability to produce IFN-*γ* in response to both specific (rhsp65) and policlonal (ConA) stimuli. In addition, splenocytes from healthy but not undernourished animals immunized with BCG produced significant levels of IFN-*γ*, TNF-*α*, and IL-10 in response to *in vitro* stimulation with rhsp65. This lower production of TNF-*α* and IFN-*γ* by undernourished mice could explain the higher BCG load in these animals, as these cytokines are known to activate macrophages to restrain mycobacterial growth [23, 24]. Similarly, lower production of these cytokines associated with undernutrition could facilitate BCG dissemination to the thymus and lungs in these animals. Even though the extent of protection afforded by BCG is highly debated, the consensus is that this vaccine is able to protect children against more severe forms of TB infections [25] and leprosy [26] and may also offer nonspecific protection against other infectious diseases [27]. Undernutrition could, therefore, severely undermine all these protective effects associated with BCG.

As we mentioned before, IL-10 production was also clearly affected by undernutrition. Whereas normal mice produced significant higher levels of this cytokine after BCG immunization, there was no IL-10 upregulation in undernourished mice. It is tempting to speculate that the immunoregulatory properties of BCG in autoimmunity and allergies are related to IL-10 production and are consequently lost in undernourished individuals. Evidence of the immunoregulatory effects of BCG in healthy animals has been described. Sewell et al. observed that *Schistosoma mansoni* OVA pretreatment, BCG infection, and lyophilized *M. tuberculosis* all decreased clinical manifestations of experimental autoimmune encephalomyelitis in mice [28]. Interestingly, this immunoregulatory activity has been associated with IL-10 production by regulatory Th17 cells [29] and dendritic cells [30].

## 5. Conclusions

Taken together, these results raise questions regarding the safety and efficacy of BCG vaccination in undernourished children. For instance, it will be important to assess whether these children develop BCGosis. Studies of the protective effects of BCG against leprosy and more severe forms of TB are also essential. Finally, the potential loss of the ability of BCG to downregulate deleterious immune responses associated with autoimmunity and allergy and the consequences of BCG dissemination to the thymus in undernourished individuals also deserve further investigation.

## Figures and Tables

**Table 1:** Physical, hematological, biochemical, and immunological parameters in undernourished BALB/c mice. Body weight was recorded daily and the other parameters were evaluated on day 10 after the beginning of dietary restriction. Values represent mean ± standard deviation of 6 animals.

Parameters	Normal *n* = 6	Restricted *n* = 6
Initial body weight (g)	20.66 ± 1.23	20.65 ± 1.15
Final body weight (g)	21.21 ± 1.16	18.68 ± 0.87^♦∗^
Total leucocytes (10^6^ cells/mL)	7.70 ± 2.54	3.95 ± 0.56*
Lymphocytes (10^6^ cells/mL)	6.89 ± 2.51	3.43 ± 0.56*
PMNs (10^6^ cells/mL)	0.78 ± 0.30	0.51 ± 0.19*
Monocytes (10^6^ cells/mL)	0.03 ± 0.01	0.01 ± 0.03*
Glucose (dL/mL)	68.25 ± 9.75	58.27 ± 7.23*
Total proteins (g/dL)	6.16 ± 0.39	6.21 ± 0.19
Albumin (g/dL)	2.52 ± 0.18	2.62 ± 0.08
Triglycerides (mg/dL)	198 ± 54	65 ± 8*
Cholesterol (mg/dL)	87 ± 9	82 ± 5
IFN-*γ* (pg/mL)	26181 ± 6589	22076 ± 3783
TNF-*α* (pg/mL)	153 ± 23	127 ± 26
IL-10 (pg/mL)	506 ± 96	487 ± 95

^♦^ *P* ≤ 0.05 compared to initial weight.

*P ≤ 0.05 compared to normal counterpart.

**Table 2:** Number of colonized BALB/c mice after BCG immunization. (A) Percentage of colonization in the lymph nodes, spleen, liver, lungs and thymus of normal immunized mice. (B) Percentage of colonization in different organs of animals immunized with BCG after 10 days of dietary restriction. (C) Percentage of colonization in different organs of household contacts (nonimmunized undernourished mice housed in conventional open cages allocated in the same room of immunized animals).

	Groups	Lymph nodes	%	Spleen	%	Liver	%	Lung	%	Thymus	%
A	Normal + BCG	7/13	54	2/6	33	1/12	8	0/7	0	0/12	0
B	Restricted + BCG	10/11	91	4/5	80	5/12	42	3/6	50	9/11	82
C	Undernourished household contacts	2/4	50	1/4	25	1/4	25	NS	NS	0/4	0

NS = not shown.
